# Separation of blood cells with differing deformability using deterministic lateral displacement^†^

**DOI:** 10.1098/rsfs.2014.0011

**Published:** 2014-12-06

**Authors:** David Holmes, Graeme Whyte, Joe Bailey, Nuria Vergara-Irigaray, Andrew Ekpenyong, Jochen Guck, Tom Duke

**Affiliations:** 1 London Centre for Nanotechnology, University College London, 17–19 Gordon Street, London WC1H 0AH, UK; 2 Friedrich-Alexander Universität Erlangen-Nürnberg, Henkestrasse 91, 91052 Erlangen, Germany; 3 Cavendish Laboratory, Department of Physics, University of Cambridge, JJ Thomson Avenue, Cambridge CB3 0HE, UK; 4 Centre for Mathematics and Physics in the Life Sciences and Experimental Biology, University College London, Gower Street, London WC1E 6BT, UK; 5 Department of Genetics, Evolution and Environment, Institute of Healthy Ageing, University College London, Gower Street, London WC1E 6BT, UK; 6 Biotechnology Center, TechnischeUniversität Dresden, Tatzberg 47/49, 01307 Dresden, Germany

**Keywords:** cell deformability, microfluidics, deterministic lateral displacement, blood separation, optical stretching, digital holography

## Abstract

Determining cell mechanical properties is increasingly recognized as a marker-free way to characterize and separate biological cells. This emerging realization has led to the development of a plethora of appropriate measurement techniques. Here, we use a fairly novel approach, deterministic lateral displacement (DLD), to separate blood cells based on their mechanical phenotype with high throughput. Human red blood cells were treated chemically to alter their membrane deformability and the effect of this alteration on the hydrodynamic behaviour of the cells in a DLD device was investigated. Cells of defined stiffness (glutaraldehyde cross-linked erythrocytes) were used to test the performance of the DLD device across a range of cell stiffness and applied shear rates. Optical stretching was used as an independent method for quantifying the variation in stiffness of the cells. Lateral displacement of cells flowing within the device, and their subsequent exit position from the device were shown to correlate with cell stiffness. Data showing how the isolation of leucocytes from whole blood varies with applied shear rate are also presented. The ability to sort leucocyte sub-populations (T-lymphocytes and neutrophils), based on a combination of cell size and deformability, demonstrates the potential for using DLD devices to perform continuous fractionation and/or enrichment of leucocyte sub-populations from whole blood.

## 1. Introduction

Cell deformability is an important emerging bio-marker for a number of disease states [1]. Deformability is indicative of underlying membrane, cytoskeletal or nuclear changes associated with a wide range of cell functional changes, such as differentiation or mitosis [2–4], or disease processes (e.g. cancer) [5–8]. For example, a reduction in erythrocyte (RBC) deformability is a contributing factor seen in many human disease pathologies and has recently been a topic of growing research interest. Diseases such as diabetes, sickle cell anaemia and malaria, as well as hereditary blood disorders such as spherocytosis, elliptocytosis and ovalocytosis all exhibit characteristic losses in RBC deformability with onset and progression of the pathological state. For the case of *Plasmodium falciparum* malaria, recent experiments have shown that the membrane stiffness of the parasitized RBC can increase more than 50-fold during intra-erythrocytic parasite maturation [9]; with malaria-infected erythrocytes showing progressive stiffening with parasite growth [10]. A reduction in stiffness has recently been identified as a potential marker in populations of pluripotent stem cells, while expression levels of the transcription factor NANOG, implicated in regulating pluripotency, have been shown to impact embryonic stem cell stiffness [11]. Leucocytes show changes in stiffness in response to activation with antigens or other stimuli [12,13], and metastatic cancer cells often show a ‘softer’ phenotype than healthy cells of the same origin [6].

An attractive benefit of cell stiffness as a bio-marker is minimal requirements for sample preparation or labelling (e.g. with magnetically or fluorescently labelled antibodies), which reduces sample preparation time and cost. It also leaves the cells in an unperturbed state, which can be important when the cells are to be used for transplantation after mechanical characterization and sorting.

The power of the mechanical phenotyping approach rests on the fact that the stiffness is largely determined by the cytoskeleton of cells, which in turn is involved in many important cell processes, such as cell polarization, migration, division, mechano-sensing or phagocytosis. Any physiological or pathological change in these functions necessarily leads to a change in the cytoskeleton and thus in cell stiffness, which can be monitored by appropriate techniques. This intimate link between cell stiffness and cell function and its implications for biotechnological and biomedical applications, as well as the inherent interest in cell biological questions, has led to the development of many different cell mechanics measurement technologies. The most prominent methods are nano-indentation with atomic force microscopy [14], micropipette aspiration [15], magnetic twisting cytometry [16], microplate deformation [17] and optical stretching [18]. All of these have particular strengths and weaknesses, but are generally marked by relatively low throughput (less than 100 cells h^−1^), which has hindered further biological and biotechnological application.

A recent development in the field of cell mechanics is the use of microfluidic approaches to facilitate sample handling and allow high-throughput probing of cell mechanical properties. A particular variant, deformability cytometry, allows the measurement of up to thousands of cells per second, but only for a few seconds [7,19]. Analysis occurs post-measurement making instantaneous sorting during the measurement impossible. The most promising microfluidic techniques rely on inertial focusing and other hydrodynamic forces generated in microfluidic channels with carefully designed geometries; these devices do allow for continuous high-throughput single-cell sorting [20].

Deterministic lateral displacement (DLD) is another powerful microfluidic technique capable of high-resolution continuous sorting of cells and other microscopic particles [21–24]. DLD devices consist of arrays of pillars positioned within a flow channel. Objects smaller than a critical size move in the direction of flow (i.e. along the channel axis) and objects larger than the critical size move in a direction defined by the pillar arrangement (i.e. they are laterally displaced). For rigid spherical objects, the operation of the device is straightforward [25]. However, biological objects (e.g. cells) are often compliant and non-spherical and their deformability and shape are known to influence the trajectories in DLD devices. Our group and others [26] have recently developed microfluidic DLD devices capable of sorting cells according to both size and deformability.

**Figure 1.**
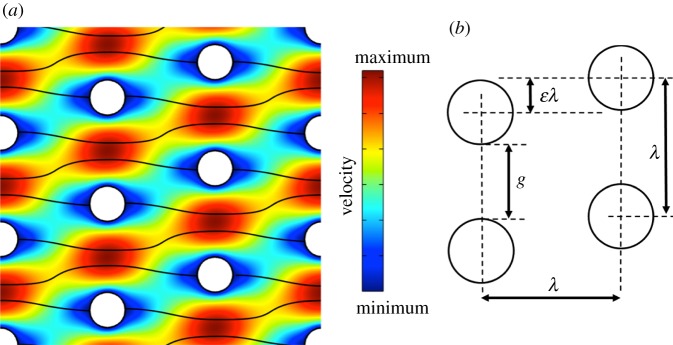
Schematic showing the fluid streamlines (at low Reynolds number) through an array of pillars. Each column is shifted vertically by *ɛλ* relative to the previous column, where *λ* is the inter-pillar distance, *ɛ* is the column shift fraction and *g* the gap between the pillars. The flow between the pillars is assumed to be parabolic with streamlines being divided by stall lines which begin and terminate on the pillars. The horizontal flow velocity is indicated by the colour intensity. (Online version in colour.)

Figure 1 shows the typical pillar arrangement found in a DLD device. An array of micrometre-sized pillars is set at an angle to the direction of fluid flow through a microfluidic channel. Figure 1*a* shows a simulation of the flow velocities through an infinite array of such pillars. As described previously [25], the total fluid flux through the gap between the pillars can be divided into a number of flow streams (drawn as black lines in the figure), each of which carries equal fluid flux. Particles flowing in the DLD device will follow different trajectories through the device depending on their size. To a first approximation particles above and below a critical size *D*_c_ will follow different paths through the array of pillars. The critical diameter being a function of the pillar diameter; the inter-pillar gap, *g*; the array period, *λ*; and the row shift fraction, *ɛ* as described in figure 1*b*. The inter column distance for the devices used in this work is also *λ*, giving a pillar lattice slope of *ɛ* to the average direction of flow.

The dynamics of rigid spherical particles flowing in DLD arrays are well understood, and the critical particle size is reasonably well described by the following equation:1.1
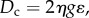
where *g* is the gap between the pillars and *η* a parameter which accounts for non-uniform parabolic flow through the gap. It is possible to continuously separate a range of particle sizes by placing arrays of different critical particle sizes in series.

The behaviour of non-spherical particles, such as erythrocytes, is less well understood and they demonstrate complex dynamic behaviour as they flow through the pillar array. Shear forces acting on non-spherical particles will tend to align these particles with their long axis along the direction of flow and presenting their short axis as the apparent size in the DLD device (e.g. the displacement properties of biconcave erythrocytes in the DLD devices will appear as that of a sphere of diameter approx. 2.7 µm). Shear-related particle orientation is a well-understood phenomenon and has been previously described for non-spherical particles flowing in DLD devices [26]. With increased flow velocity comes higher shear forces and particle deformation becomes an issue of increased importance, further complicating the dynamic behaviour of particles in these systems. Particles therefore present an ‘apparent size’ to the DLD device, which is a result of both shear-related orientation and deformation.

In this work, we present characterization of deformability-based cell separation in a DLD device. Cells of defined stiffness (glutaraldehyde cross-linked erythrocytes) are used to test the performance of the device. Optical stretching and digital holography are used to independently measure differences in the viscoelastic properties of these cells, thus allowing better understanding of the DLD system for deformability-based cell separation. We also demonstrate the effect of shear rate on the lateral displacement of leucocyte sub-populations within similar devices and show our work towards continuous fractionation of whole blood leucocyte populations.

## 2. Material and methods

### 2.1. Cell samples and buffers

Blood samples were obtained via fingerprick: 50 µl of whole blood was collected and the cells washed three times in 2 ml of isotonic phosphate buffered saline (PBS) solution (0.01 M phosphate, 0.137 M NaCl, 0.0027 M KCl) containing 2 mM EDTA.

### 2.2. Treatment of erythrocytes with glutaraldehyde

Blood cells were resuspended at haematocrit of 2.5% by volume in the required concentrations of glutaraldehyde (0–0.01% in PBS) and incubated at room temperature for 45 min. The samples were again washed three times in PBS and resuspended in running buffer (PBS with 0.1% w/v Pluronic F108, Sigma, UK). A final cell density of 1 × 10^5^ cells ml^−1^ was used in all experiments.

### 2.3. Fluorescent labelling of leucocytes

Fifty microlitres of whole blood was diluted into 1 ml of PBS (1 : 20 v/v) containing 2 mM EDTA. One microlitre of 1 mM CellTracker dye (CMFDA, Molecular Probes) was then added to the sample. The sample was incubated for 10 min at room temperature and then run through the device, without further washing of the sample.

### 2.4. Fluorescent labelling of leucocyte sub-populations

Fifty microlitres of whole blood was incubated with fluorescently conjugated monoclonal antibodies directed against CD3 (Alexa647) and CD16 (Alexa488) (both from BioLegend, San Diego, CA, USA) for 15 min in the dark at 4°C, to fluorescently label the T-lymphocyte and neutrophil populations, respectively. The entire blood sample was then diluted into 1 ml of PBS (1 : 20 v/v) containing 2 mM EDTA and run through the device.

### 2.5. Microfluidic device fabrication

Two microfluidic device geometries were used throughout this work: (i) a shallow, 4.5 µm channel height, geometry was used for the experiments involving erythrocytes as this device confined the erythrocytes (reduced the number of degrees of freedom) ensuring they flow through the device presenting their major axis perpendicular to the direction of flow within the DLD device and (ii) a deeper, 25 µm channel height, geometry that allowed the erythrocytes to align with the flow (present their minor axis perpendicular to the direction of flow) and pass through the DLD device without lateral displacement. The microfluidic devices comprised polydimethylsiloxane (PDMS) channel structures bonded to glass microscope slides. Channel designs were produced in AutoCAD. Standard photolithography techniques were used to produce a silicon (Si) master from which PDMS channel structures were cast. The process is summarized in the following: a chrome mask (JD Photo Tools, UK) was used to pattern a 1.8 µm thick layer of positive tone photoresist (Microposit S1818, Shipley, UK) spun onto a 100 mm Si wafer (Compart Technology Ltd, Tamworth, UK). The exposed photoresist was developed using Microposit MF319 (Shipley, UK) and hard-baked on a hotplate at 115°C for 5 min. The wafer was then dry etched using an STS-DRIE system running the Bosch process, with the patterned photoresist acting as a protective etch mask. Following etching, the photoresist was removed in acetone leaving the patterned wafer. The Si wafer was passivated by immersing for 10 min in a 1% solution of tridecafluoro(1,1,2,2-tetrahydrooctyl)trichlorosilane in toluene; this step allowed subsequent release of cured PDMS from the Si master. A 10 : 1 mixture of degassed PDMS was poured onto the wafer and baked overnight at 65°C until fully cured. Individual PDMS devices were then cut from the cured PDMS block and inlet and outlet holes punched using a biopsy punch (Technical Innovations, USA). The PDMS structures and the glass slides were both rinsed in isopropyl alcohol and blown dry prior to plasma treatment using a BD-20 Laboratory Corona Treater (Electro-Technic Products Inc., Chicago, IL, USA). Glass and PDMS were then brought into contact within a few seconds of treatment and left in an oven overnight at 65°C to fully bond.

### 2.6. Experimental set-up

The microfluidic chips were mounted on a fluorescence microscope (Nikon Eclipse) to allow imaging of cells passing through the device. One of two cameras was used depending on the requirement of the experimental observation: either an Orca-ER (Hamamatsu) or a high-speed image intensified camera (Focuscope SV-200, Photron). Image processing was carried out using ImageJ and bespoke image analysis code written in Mathematica. Buffers and cell samples were introduced into the device under the control of a multi-channel flow controller (MFCS-8C, Fluigent), which allowed accurate control of the applied pressure between 0 and 1300 mbar. Sample reservoirs were connected to the chip using small bore Tygon tubing (1/16″ outer diameter × 0.51 mm inner diameter) and short sections of 23G stainless steel tube (Elveflow, Paris, France). The camera and pump were both controlled via PC and in-house written LabVIEW code.

### 2.7. Sample introduction and channel priming

The microfluidic device was first primed with a 0.5% (w/v) solution of Pluronic F-108 surfactant in PBS. The wetting properties of the Pluronic solution allowed for easy filling of the microfluidic channels and purging of air bubbles. Following channel filling, the Pluronic was allowed to incubate for a minimum of 20 min in order to block the PDMS and glass surfaces from further hydrophobic and other non-specific adhesive interactions with the red cell. The system was then flushed with 1 ml of PBS containing 1% (w/v) bovine serum albumin. Cell samples were then introduced to the channel sample inlet and running buffer was introduced via the adjacent buffer inlet channels. Outlet channels were held at atmospheric pressure.

### 2.8. Optical stretcher

Optical trapping and stretching of erythrocytes was performed using a bespoke optical stretcher. The optical stretcher is an established technique for measuring the mechanical properties of cells in suspension using optical fields. The system used was similar to previously described systems [27,28] and will therefore only be briefly described here. The optical stretcher consists of a dual-beam fibre-optical trap aligned perpendicularly to a microfluidic flow channel (square cross-section glass capillary). The optical fibres and flow channel were aligned and held in place using a micro alignment structure fabricated photolithographically from SU8 on a glass substrate. When running cells can be stably trapped, in the optical stretcher, from a flowing fluid at a low optical power (*ca* 100 mW) and held in position. If a higher power is used, the optical forces at the surface of the cell, which are concentrated on the beam axis, become large enough to measurably deform the cell. The forces exerted on the cell depend on the laser power, the arrangement of the trap and the refractive index of the cell and suspending medium. For most cell types, the optical forces being exerted [29,30] can be calculated, which can give the compliance from the measured uniaxial deformation. In our set-up, the light power in both beams was held at 100 mW allowing trapping of individual cells. When transiently increased to 600 mW, the increased radiation pressure causes the cell to deform (‘stretch’).

## 3. Results and discussion

**Figure 2.**
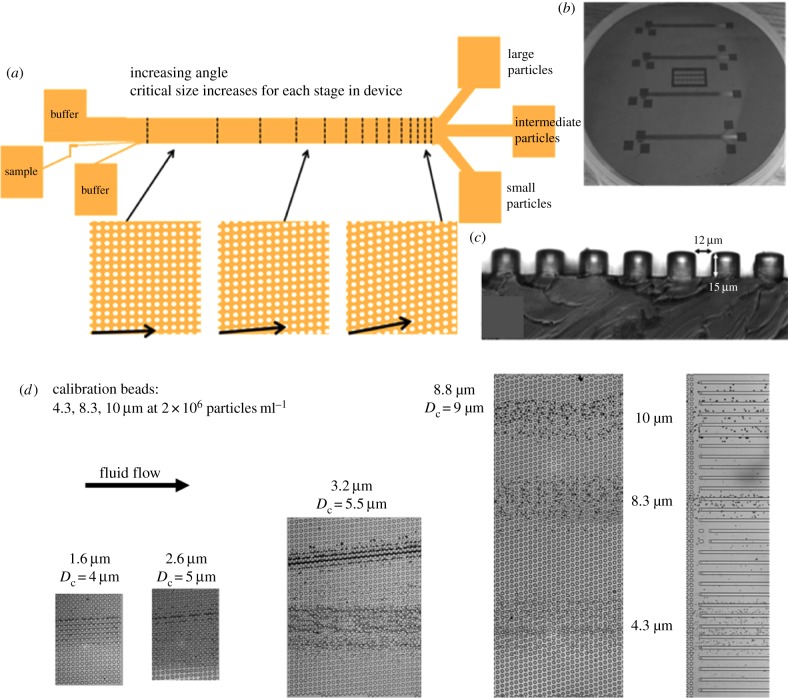
(*a*) Schematic of DLD separation device showing multiple sections each designed to achieve a characteristic critical diameter. (*b*) DRIE etched silicon master for casting of PDMS devices. (*c*) Section through a PDMS device showing the pillars. Depending on the cell type under study, the height of the pillars was varied: approximately 4 µm tall pillars for erythrocyte work and approximately 25 µm for experiments involving whole blood. (*d*) Micrographs showing the trajectories of a mixture of rigid polymer beads (i.e. non-deformable particles) at different positions along the DLD device. The numbers by each image correspond to the vertical column to column shift, and the associated critical diameter for that particular section of the DLD device. (Online version in colour.)

Figure 2*a* shows a schematic of the DLD separation devices used throughout this work. The device comprises multiple sections, each with a characteristic critical diameter. Each section of the device is designed to laterally displace particles with a size greater than *D*_c_ by 200 µm. The first section of the device has *D*_c_ = 3 µm and each section has a progressively larger value of *D*_c_ increasing by 0.5 µm with each subsequent section. Figure 2*b*,*c* shows the etched silicon master for casting of PDMS devices and a cut section through a PDMS device clearly showing the vertical pillar profiles.

### 3.1. Deterministic lateral displacement of hard spherical objects

In order to assess the performance of the DLD device, a mixed sample of polymer beads was run through the device. Typical particle velocities are less than 1 mm s^−1^, and as such inertial effects are negligible with the Reynolds number of the order of 10^−2^. Figure 2*d* shows the trajectories of rigid polymer beads (i.e. non-deformable particles) at different positions along the DLD device. A mix of 4.3, 8.3 and 10 µm latex beads was introduced to the device and imaged at various positions along the device. Clear separation of the different size beads is observed at the outlet and other positions along the length of the device.

### 3.2. Increased glutaraldehyde concentration confers increased stiffness to erythrocytes

Human erythrocytes were treated with varying concentrations of glutaraldehyde to modify the stiffness of the cells. The glutaraldehyde acts through cross-linking of the cellular proteins specifically those of the spectrin network and associated proteins [31]. A highly fixed cell is essentially a discoid-shaped non-deformable rigid body (i.e. an extreme in terms of membrane rigidity). Cells exposed to fixation under less severe conditions (i.e. lower concentrations of glutaraldehyde) showed differing degrees of fixation and different levels of stiffness. Glutaraldehyde concentrations in the cross-linking solution were varied from 0% up to 0.1%. Samples of cells were treated similarly and measured using the optical stretcher and on the DLD cell fractionation device. No observable differences were seen in the morphology of the glutaraldehyde-treated and untreated erythrocytes; this is supported by our observations and in agreement with previous work using similar treatment conditions [31].

### 3.3. Optical stretching correlates with erythrocyte stiffness

**Figure 3.**
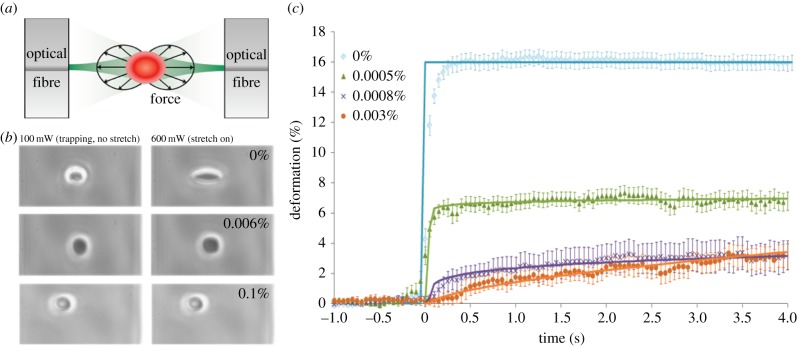
(*a*) Schematic of the optical stretching set-up. (*b*) Microscope images of trapped erythrocytes held under 100 mW power (left) and stretched erythrocytes under 600 mW power (right). Each row of images shows cells having been exposed to different glutaraldehyde concentrations (concentration is noted in the right upper corner of each panel). (*c*) Average stretching curves for erythrocytes with various levels of glutaraldehyde cross-linking (*n* > 20 for each concentration). Solid lines represent power law fits to the data. Error bars show standard error of mean. (Online version in colour.)

Figure 3*a* shows a schematic of the optical stretcher set-up and a cell undergoing stretching. Treatment of cells with different concentrations of glutaraldehyde resulted in well-defined increases in cell stiffness. Figure 3*b* shows images from the cell stretching experiments for a range of different concentrations of glutaraldehyde. The optical stretcher data showed a monotonic increase in cell stiffness with increasing glutaraldehyde concentration up to concentrations of approximately 0.02%. Figure 3*c* shows stretch data up to 0.003% glutaraldehyde concentration. Above this concentration, the erythrocytes no longer deform under the applied forces achievable using the optical stretcher. This progressive increase in cell stiffness correlates well with data from other groups, where very large increases in stiffness were seen with glutaraldehyde concentrations in the range between 0 and 0.025% [31]. Quantitative phase measurements, using digital holographic microscopy (see the electronic supplementary material) showed a negligible increase in refractive index of cells treated with glutaraldehyde; even extremely high levels (0.1%). Glutaraldehyde treatment introduced less than 1% bias into the stretch measurement across the concentration range used in this work. Changes in the optical properties of the cells following treatment with glutaraldehyde can therefore have only negligible influence on the magnitude of the cell stretching observed. The degree of cell stretching is therefore taken to be a good measure of the stiffness of the measured erythrocytes. As the shape and refractive indices of the erythrocytes remained the same for the different conditions (i.e. across the range of glutaraldehyde concentrations), the compliance of the cells can be assumed to be directly proportional to the measured optically induced deformations.

### 3.4. Separation of erythrocytes of different stiffness using deterministic lateral displacement

**Figure 4.**
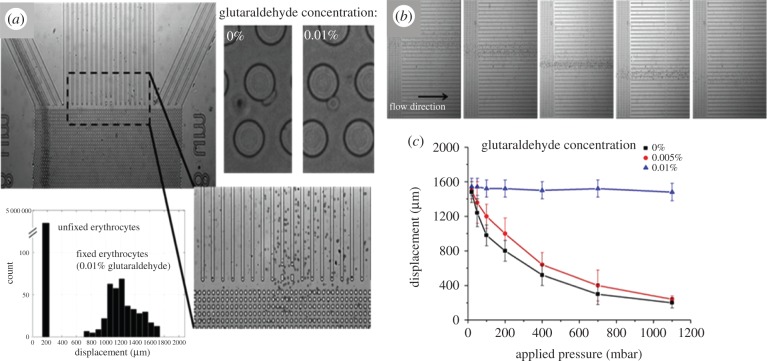
(*a*) Photograph of the outlet of the shallow DLD device showing separation of RBCs of different stiffness (mixture of 0 and 0.01% glutaraldehyde-fixed erythrocytes). Histogram shows cell distribution at the outlet for a mix of untreated and glutaraldehyde-treated RBCs (note that the displacement direction is reversed with respect to figures 1 and 2). The high-magnification images (top right) show deformation of compliant (0% glutaraldehyde) and stiff (0.01% glutaraldehyde) erythrocytes as they interact with the pillars within the DLD device. (*b*) Photographs of the outlet of the DLD device showing displacement of untreated erythrocytes at different flow rates (increasing flow rates are shown from left to right). (*c*) Variation in lateral displacement versus flow rate for erythrocytes of different stiffness. (Online version in colour.)

To assess the effect of stiffness on the hydrodynamic trajectories of the cells flowing though the DLD device, samples of untreated and glutaraldehyde-treated erythrocytes were run through the DLD device under a range of flow conditions (i.e. varying levels of shear stress). Video images of the cells within the device and their distribution across the width of outlet of the device were recorded. Samples of defined stiffness (i.e. defined concentration of glutaraldehyde) were run through the device as well as mixture of cells having differing stiffness (i.e. mixtures of cells which were exposed to different glutaraldehyde concentrations and subsequently washed and mixed together in known concentrations). Figure 4*a* shows a microscope image of the outlet of the DLD device. The data in the image and histogram are from an experiment where a mixture of 0 and 0.01% glutaraldehyde-fixed erythrocytes was run through the device with an applied pressure of 1000 mbar. The histogram shows the cell distribution at the outlet of the DLD device (it should be noted that the displacement direction across the channel is reversed with respect to that of figures 1 and 2). The high-magnification images (top right, figure 4*a*) show high-speed video images of the deformation of compliant (0% glutaraldehyde) and stiff (0.01% glutaraldehyde) erythrocytes as they interact with the pillars within the DLD device. Figure 4*b* shows the displacement of untreated erythrocytes under a range of different flow conditions. Progressive images show the position of the erythrocytes for increasing levels of shear rate (shear rate increasing as images progress from left to right). The data in figure 4*c* show the average lateral displacement at the outlet of the DLD device, for three erythrocyte populations of clearly defined stiffness across a range of applied shear rates.

### 3.5. Separation of leucocytes using deterministic lateral displacement

**Figure 5.**
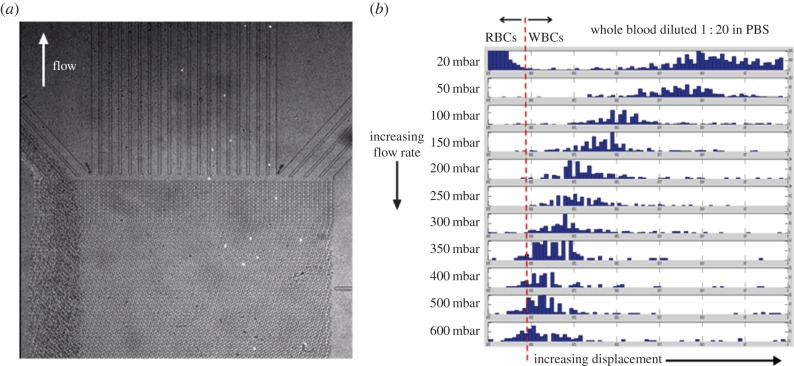
(*a*) Microscope image showing separation of leucocytes (bright labelled cells) from whole blood using the deep DLD device. Leucocytes are labelled with CellTracker dye. Erythrocytes are not displaced (the greater depth of the device means they are free to orient along the direction of fluid flow) and run straight through to the outlet at the top left. (*b*) Lateral displacement of leucocytes (WBCs) is shown as a function of flow rate. Lateral displacement is seen to decrease as the flow rate is increased; this is due to cells deforming under shear. (Online version in colour.)

The effect of shear rate on the separation of human leucocytes was also investigated, again using washed whole blood. Similar devices to those described above, for the erythrocyte work, were used to study the separation of leucocytes from 1 : 20 diluted whole blood. For this work, the DLD device channel depth was approximately 25 µm. This channel geometry allows the RBCs more degrees of rotational freedom and these cells tend to align with their minor axis presenting across the inter-pillar gap. For this pillar gap size and flow condition used, the RBCs experience no lateral displacement within these taller devices. The leucocyte population does, however, experience a lateral displacement in these devices, which is seen to vary with shear rate. This variation in the lateral displacement with shear rate is a result of the cells distorting in the fluid; this has been well documented in DLD devices [19] and other microfluidic flow regimes [23] and could clearly be seen in the DLD devices used in this work (data not shown). As a result, as the leucocytes flow through the device their apparent size decreases with increasing shear. Figure 5*a* shows a fluorescence image of the outlet of the DLD device, the leucocytes have been labelled with a fluorescent CellTracker dye to allow visualization and can be seen as bright objects towards the right-hand side of the image. The erythrocytes all flow to the leftmost outlet of the device. The histograms in figure 5*b* show the characteristic distribution of the leucocyte population at the outlet of the device as a function of applied pressure at the channel inlet (with the outlet held at atmospheric pressure). The leucocyte displacement follows a similar relaxation in the magnitude of lateral displacement as that of the RBCs (as seen in the shallow device geometry, described above). This reduction in the lateral displacement with increased shear rate is again attributed to the distortion of the cell shape due to shear stress on the cells as they pass through the device and interact with the pillars.

**Figure 6.**
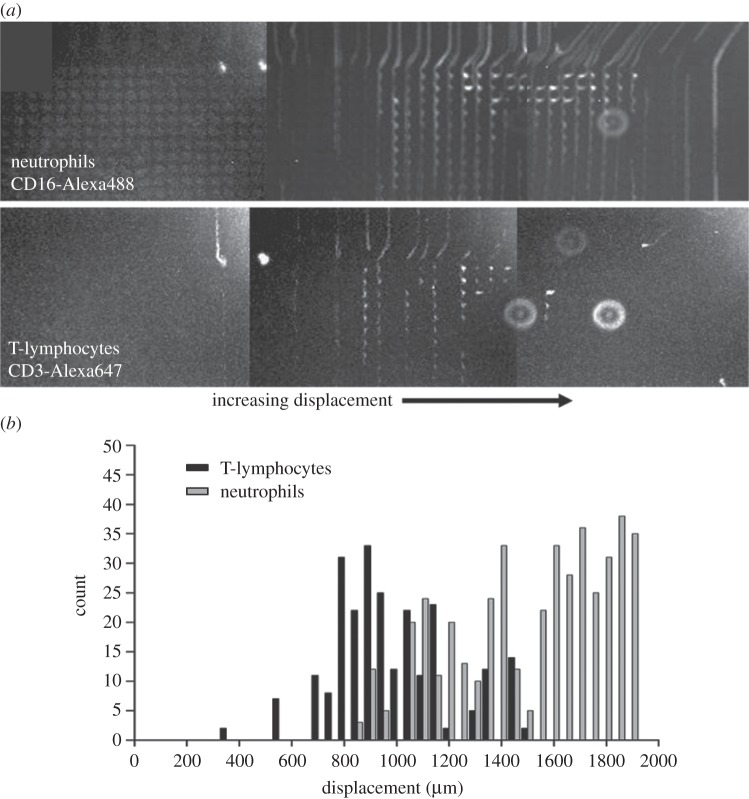
(*a*) Images of fluorescently labelled leucocyte sub-populations (in diluted whole blood) flowing through the outlet of the deep DLD device. T-lymphocytes and neutrophils clearly exit the device with different displacement distributions. The bright vertical lines in the image are created from superimposition of multiple video frames; each image shows several hundred cells superimposed. (*b*) Histogram showing the lateral distribution of the T-lymphocyte and neutrophil populations at the outlet of the device.

With increased shear rate, the leucocyte distributions, as a whole, clearly show less lateral displacement. However, due to the heterogeneous mixture of cell types making up the leucocyte population in whole blood, one might expect that the changing cell distributions contain regions (i.e. certain lateral displacements) that show enrichment or depletion of certain sub-populations of the leucocyte population. It should be noted that any change in the apparent size of the cells due to shear as they flow through the device will be a combination of both cell size (the average size of the leucocyte cell populations differs among the cell types) and the deformability of the cell. To this end, we performed experiments using 1 : 20 PBS diluted whole blood that had the T-lymphocyte and neutrophil populations fluorescently labelled with different fluorophores to allow simultaneous imaging of the distribution of these sub-populations of cells at the outlet of the DLD device. Figure 6*a* shows multiple superimposed video images of the labelled cells flowing through the outlet of the device. At low shear rates (applied pressure 20 mbar), all the leucocytes within the sample were seen to be displaced laterally across the majority of the width of the device and exited in a band close to the rightmost channel wall of the device. At higher shear rates (*ca* 200 mbar), the leucocytes are seen to undergo deformation as they flow through the device. As described above, this deformation results in an apparent reduction of the cell size within the device, resulting in a reduction in the lateral displacement seen at the outlet of the device. Under certain conditions of flow (i.e. appropriately chosen shear rates), it can be demonstrated that sub-populations of the leucocytes exit the device with differing degrees of lateral displacement, thus showing the potential for the development of a continuous leucocyte fractionation device. The histogram shown in figure 6*b* shows the distributions of T-lymphocytes (labelled with CD3-Alexa647) and neutrophils (labelled with CD16-Alexa488) at the outlet of the DLD device. Two clear distributions can be seen in the histograms. The populations show some overlap, but careful design of the outlet ports of the device should allow continuous fractionation of leucocyte sub-populations under appropriate flow conditions.

## 4. Conclusion

We have demonstrated the effect of cell stiffness on separation in a DLD device and have shown stiffness to be a useful parameter for separation in such devices. Erythrocytes treated within a range of concentrations of glutaraldehyde (0–0.01%) show a near linear increase in cell stiffness with concentration. Through the use of optical stretching, we measured the relative stiffness of different cell populations and we are able to show for the first time a direct correlation between cell stiffness and lateral displacement in a DLD device.

The stiffness of the chemically modified RBCs used in this work is similar to that of erythrocytes infected with the malaria parasite *P. falciparum* (previously measured via optical stretching [32]), thus demonstrating the potential of using the DLD technique as a method for isolating malaria-infected cells from blood. We are currently continuing this work and further developing the DLD technique, in collaboration with colleagues at the London School of Hygiene and Tropical Medicine with the aim of producing an integrated device for isolation of malaria-infected cells from blood samples.

The additional demonstration that leucocytes, and even sub-populations of leucocytes (T-lymphocytes and neutrophils), can be separated both from RBCs as well as from each other, based on a combination of both size and deformability, opens potentially new avenues for the use of DLD devices. So far, whole blood fractionation requires tedious handling involving density-gradient centrifugation and manual pipetting; or magnetic separation with appropriate antibodies. Using DLD, the continuous label-free sorting of blood cells becomes possible, reducing preparation time, cost and effort, while leaving the cells in their native state. With technological advances such as described in this paper, the promise of cell mechanics for characterization and sorting of cell populations is finally moving towards widespread application in biology, biotechnology and medicine.
